# Spinal muscular atrophy

**DOI:** 10.1186/1750-1172-6-71

**Published:** 2011-11-02

**Authors:** Adele D'Amico, Eugenio Mercuri, Francesco D Tiziano, Enrico Bertini

**Affiliations:** 1 Department of Neurosciences, Unit of Molecular Medicine for Neuromuscular and Neurodegenerative Disorders, Bambino Gesu' Children's Research Hospital, P.za S. Onofrio, 4, Rome (00165), Italy; 2 Dept of Neurology, Unit of Pediatric Neurology, Catholic University, Largo F. Vito 1, Rome (00168), Italy; 3 Laboratory of Cytogenetics and Molecular Biology, Institute of Medical Genetics, Catholic University, Largo F. Vito 1, Rome (00168), Italy

**Keywords:** Proximal spinal muscular atrophy, SMN1, SMN2, motor neurons Disease names and synonyms: Spinal muscular atrophy 5q linked, Proximal SMA

## Abstract

Spinal muscular atrophy (SMA) is an autosomal recessive neuromuscular disease characterized by degeneration of alpha motor neurons in the spinal cord, resulting in progressive proximal muscle weakness and paralysis. Estimated incidence is 1 in 6,000 to 1 in 10,000 live births and carrier frequency of 1/40-1/60. This disease is characterized by generalized muscle weakness and atrophy predominating in proximal limb muscles, and phenotype is classified into four grades of severity (SMA I, SMAII, SMAIII, SMA IV) based on age of onset and motor function achieved. This disease is caused by homozygous mutations of the survival motor neuron 1 (*SMN1*) gene, and the diagnostic test demonstrates in most patients the homozygous deletion of the *SMN1 *gene, generally showing the absence of *SMN1 *exon 7. The test achieves up to 95% sensitivity and nearly 100% specificity. Differential diagnosis should be considered with other neuromuscular disorders which are not associated with increased CK manifesting as infantile hypotonia or as limb girdle weakness starting later in life.

Considering the high carrier frequency, carrier testing is requested by siblings of patients or of parents of SMA children and are aimed at gaining information that may help with reproductive planning. Individuals at risk should be tested first and, in case of testing positive, the partner should be then analyzed. It is recommended that in case of a request on carrier testing on siblings of an affected SMA infant, a detailed neurological examination should be done and consideration given doing the direct test to exclude SMA. Prenatal diagnosis should be offered to couples who have previously had a child affected with SMA (recurrence risk 25%). The role of follow-up coordination has to be managed by an expert in neuromuscular disorders and in SMA who is able to plan a multidisciplinary intervention that includes pulmonary, gastroenterology/nutrition, and orthopedic care. Prognosis depends on the phenotypic severity going from high mortality within the first year for SMA type 1 to no mortality for the chronic and later onset forms.

## Definition

Spinal muscular atrophy (SMA) is a severe neuromuscular disease characterized by degeneration of alpha motor neurons in the spinal cord, resulting in progressive proximal muscle weakness and paralysis. The disease was first described in the 1890s by Werdnig [1] and by Hoffmann [2]. The genetic defect was localized to 5q11.2-q13.3 a century later [3] with the identification of the survival motor neuron gene (SMN) gene as the disease-causing gene in 1995 [4].

## Epidemiology

SMA is the second most common fatal autosomal recessive disorder after cystic fibrosis, with an estimated incidence of 1 in 6,000 to 1 in 10,000 live births, with a carrier frequency of 1/40-1/60 [5,6].

## Clinical description and Classification

SMA is clinical classified into four phenotypes on the basis of age of onset and motor function achieved [7] (See table 1).

**Table 1:** Clinical classification criteria for spinal muscular atrophy

	Age of onset	Highest function achieved
**Type I (Werdnig-Hoffmann disease)**	0-6 months	Never sit
**Type II (intermediate)**	7-18 months	Sit never stand
**Type III (mild, Kugelberg-Welander disease) in adulthood**	> 18 months	Stand and Walk during aldulthood
**Type IV (adult)**	2°-3° decade	Walk unaided

**SMA type 1 **(Werdnig-Hoffmann disease) is the most severe and common type, which accounts for about 50% of patients diagnosed with SMA. Classically infants with SMA type I have onset of clinical signs before 6 months of age, never acquire the ability to sit unsupported and, if no intervention is provided, generally do not survive beyond the first 2 years. These patients have profound hypotonia, symmetrical flaccid paralysis, and often no head control. Spontaneous motility is generally poor and antigravity movements of limbs are not typically observed. In the most severe forms decreased intrauterine movements suggest prenatal onset of the disease and present with severe weakness and joint contractures at birth and has been labeled SMN 0. Some of these children may show also congenital bone fractures and extremely thin ribs [8-11].

Within SMA type I at least 3 clinical subgroups can be defined according to the severity of clinical signs: a) severe weakness since birth/neonatal period, head control is never achieved; b) onset of weakness after the neonatal period but generally within 2 months, head control is never achieved; c) onset of weakness after the neonatal period but head control is achieved. Some of these children may be able to sit with support [12].

Clinically, all children with SMA type I show a combination of severe hypotonia and weakness, with sparing of the facial muscles, invariably associated with a typical respiratory pattern. The weakness is usually symmetrical and more proximal than distal, with lower limbs generally weaker than upper limbs. Deep tendon reflexes are absent or diminished but sensitivity is preserved.

The spared diaphragm, combined with weakened intercostal muscles, results in paradoxical breathing. The involvement of bulbar motorneurons often give tongue fasciculation, poor suck and swallow with increasing swallowing and feeding difficulty over time. Aspiration pneumonia is an important cause of morbidity and mortality.

In the last few years there has been increasing evidence that some cases with severe SMA type I (generally carrying 1 copy of SMN2) may have heart defects [13,14], mostly atrial and ventricular septal defects and a possible involvement of the autonomic system that may be responsible for arrhythmia and sudden death.

**SMA type II **is characterized by onset between 7 and 18 months of age. Patients achieve the ability to sit unsupported and some of them are able to acquire standing position, but they do not acquire the ability to walk independently. Deep tendon reflexes are absent and fine tremors of upper extremities are common. Joint contractures and kyphoscoliosis are very common and can occur in the first years of life in the more severe type II patients. Weak swallowing can be present but is not common [15] while weakness of the masticatory muscles more often affect the ability to chew. There is a spectrum of severity ranging from weak children who are just able to sit unsupported and are more prone to respiratory signs and early scoliosis to relatively stronger children who have much stronger trunk, limb and respiratory muscles. Patients at the weak end of the spectrum may develop respiratory failure requiring mechanical ventilation.

**SMA type III **(Kugelberg-Welander disease) includes clinically heterogeneous patients. They typically reach all major motor milestones, as well as independent walking. However during infancy they develop proximal muscular weakness. Some might need wheelchair assistance in childhood, whereas others might continue to walk and live productive adult lives with minor muscular weakness. Patients who lose ambulation often develop scoliosis and other medical problems related to poor mobility such obesity and osteoporosis [16-18]. Concerning natural history data on 329 SMA type III patients, 2 subgroups of severity have been suggested on the probability of being able to walk by 10 years and on increased probability to lose walking by the age of 40 years. Significant differences loosing ability to walk were observed in relation to those with an onset of weakness before (SMA III a) and after age 3 years of age (SMA IIIb) [19].

SMA type IV has been added to this classification to describe those patients with adult onset (> 18 ys) and mild course. This group includes patients who are able to walk in adulthood and without respiratory and nutritional problems.

Since all SMA types belong to a single spectrum and share the same etiology, patient selection for clinical trials is actually independent of the historical classification, and is essentially determined by the intervention characteristics and the choice of endpoints.

## Molecular genetics and Etiology

Two almost identical *SMN *genes are present on chromosome 5q13: the telomeric or *SMN1 *gene, which is the spinal muscular atrophy- determining gene, and the centromeric or *SMN2 *gene.

The coding sequence of *SMN2 *differs from that of *SMN1 *by a single nucleotide (840C > T), which does not alter the aminoacidic sequence but results in alternative splicing of exon 7. Due to the alternative splicing of exon 7, *SMN2 *genes produce a reduced amount of full length transcripts (SMN-fl) and protein, and a variable amount of mRNA lacking exon 7 (10% to 50%, SMN-del7) which give raise to a truncated and unstable protein [20]. About 95% of patients have a homozygous disruption of *SMN1 *due to deletion or gene conversion of *SMN1 *to *SMN2 *[21]. About 3% of affected individuals are compound heterozygotes for deletion of one *SMN1 *allele and subtle intragenic mutations. All patients, however, retain at least one copy of *SMN2*, generally 2-4. Loss of *SMN1 *is essential to the pathogenesis of SMA, while the severity of the disease is primarily related to the number of copies of *SMN2*. Most SMA type I patients have two copies of *SMN2 *[22], three *SMN2 *copies are common in SMA type II, while type III and IV generally have three or four [23,24].

*SMN *genes encode for SMN protein which is ubiquitously expressed and localized in the cytoplasm and in the nucleus, and is particularly abundant in motor neurons of the spinal cord [25]. Within the nucleus, SMN protein is concentrated in dot-like structures associated with coiled (Cajal) bodies, named "gems" (gemini of coiled bodies) [26]. Although the exact cellular function of SMN protein responsible for the pathogenesis of SMA remains unknown, cells from patients with spinal muscular atrophy contain fewer gems compared controls and carriers [26].

Animal models by disruption of *SMN *has been obtained in yeast, nematode, fly, zebrafish, and mouse. These models of spinal muscular atrophy have not only been fundamental for increasing knowledge about the molecular and cellular pathways of SMN, but also to better understand the mechanism(s) of disease, and to provide a platform from which high-throughput genetic and drug screens can be performed [27]. However Spinal muscular atrophy mutant mice that die soon after birth (low copy *SMN2*+*/*+*;Smn -/-*) preclude detailed analysis of pathogenic mechanisms and preclinical drug testing [28] However, this disease severity can be tempered to intermediate and mild phenotypes by adding additional transgenes that express various wild-type isoforms or weak mutant forms of SMN [29].

Two main hypothesis have been postulated to explain the pathogenesis of SMA: (a) SMN is involved in the biogenesis of small nuclear ribonucleoproteins (snRNPs) and in mRNA splicing: thus SMN reduction may determine a general perturbation in snRNP assembly (to which motor neurons may be more sensitive), and/or SMN complex is involved in the splicing of one or few transcripts with a key function in motor neurons; or (b) SMN has a motor neuron specific function, independent from snRNPs assembly, such as mRNA transport along the axon.

Hypothesis (a) is supported by different experimental evidences: SMN protein is a part of a high molecular weight complex including at least eight other proteins, and it is necessary for proper assembly of Smith class core proteins in the Uridine-rich snRNPs (U snRNP). U snRNPs are the principal components of spliceosomes, the cellular particles that executes pre-mRNA splicing. Although SMN protein is expressed in all somatic cells, why motor neurons of the spinal cord are specifically vulnerable in spinal muscular atrophy is puzzling. Some studies suggest that SMN protein might play a key role in cellular functions unique to motor neurons [30-32].

Also hypothesis (b) is supported by different lines of evidence: several studies suggest that SMN protein might sustain the survival of motor neurons by allowing normal axonal transport and maintaining the integrity of neuromuscular junctions. Low concentrations of SMN protein might be specifically detrimental to motor neurons due to the length of axons and to their unique interactions with skeletal muscles [33-40]. Furthermore, SMN protein is localized in ribonucleoprotein granules in neurites and growth cones of motor neurons; for this reason some Authors suggested that SMN protein might be involved in transportation of ribonucleoprotein complexes containing β-actin, and/or specific mRNAs [41]. Very recently, in a mouse model of SMA it has been observed that morphological changes occurring at early stages of the disease, include reduced proprioceptive reflexes that correlate with decreased number and function of synapses on motor neuron somata and proximal dendrites. These changes occur first in motor neurons innervating proximal hindlimb muscles and in medial motor neurons innervating axial muscles. At an end-stage disease deafferentation of motor neurons occur for motor neurons innervating distal hind limb muscles. Motor neuron loss follows afferent synapse loss with the same temporal and topographical pattern [42].

## Diagnosis

Clinical features are highly suggestive for the diagnosis of SMA particularly in the severe variant of a floppy baby or weak child. The attentiveness and intellect is always good. The weakness is usually symmetrical and more proximal than distal; generally it is greater in the legs than in the arms. The severity of weakness correlates with the age of onset with delayed motor milestones according to clinical classification (see table 1). Sensitivity is preserved and deep tendon reflexes are more or less involved depending on age at onset and duration of the disease. In the most severe form moreover other clinical features include: impaired head control, weak cry and cough, swallowing and feeding difficulty, atrophy and fasciculation of the tongue and the infant relies on the diaphragm for breathing (abdominal breathing)..

**Figure 1:**
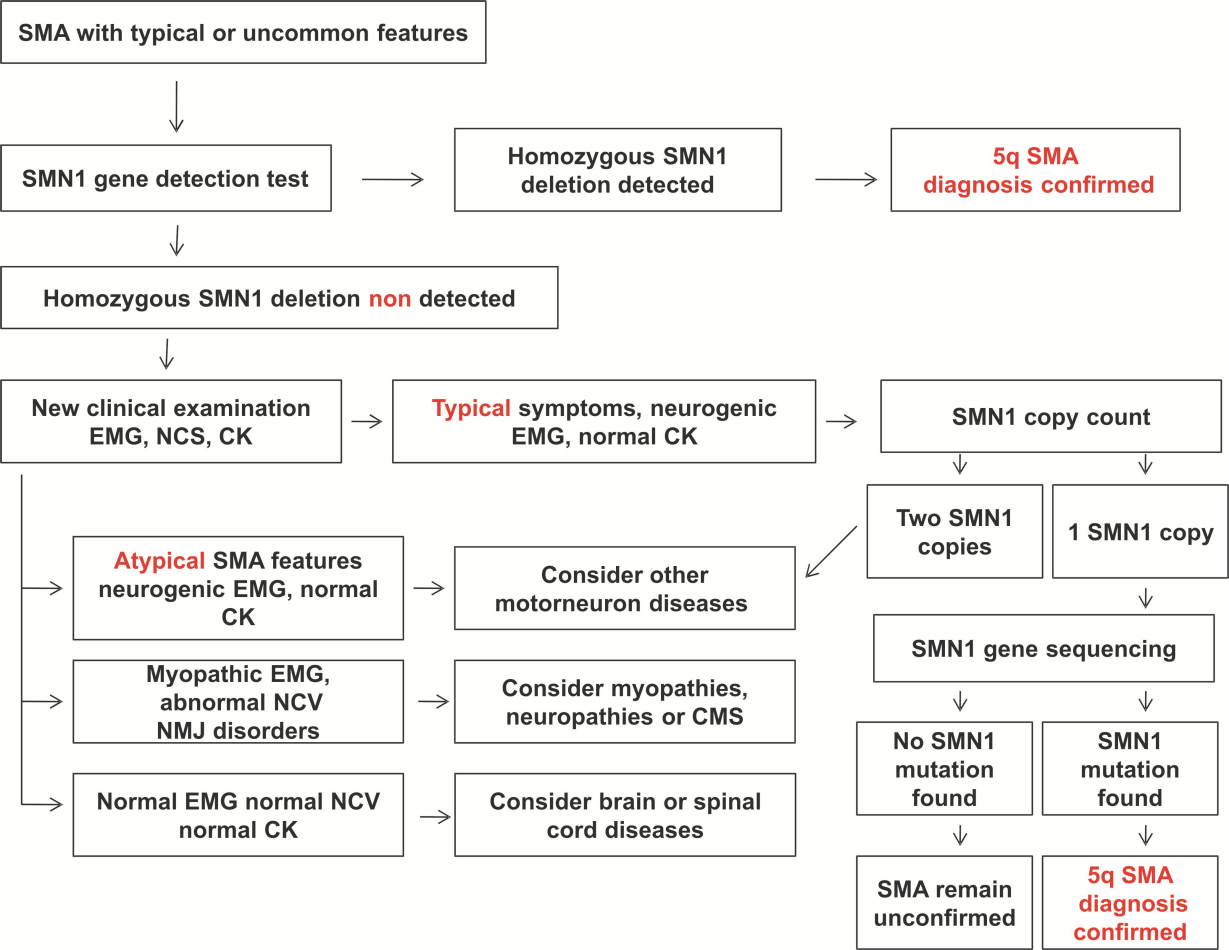
**Diagnostic algorithm for spinal muscular atrophy**.

The algorithm of the diagnostic procedures that should be guide to diagnosis of SMA is summarized in Figure 1.

The first level diagnostic test for a patient suspected to have SMA should be the search of *SMN1 *gene homozygous deletion. The absence of *SMN1 *exon 7 (with or without deletion of exon 8) confirms the diagnosis of SMA. The test achieves up to 95% sensitivity and nearly 100% specificity [43].

If the first level assay tests negative, further laboratory exams including creatine kinases dosage and electrophysiological tests such as electromyography (EMG), and nerve conduction study should be performed. If EMG suggests a motor neuron disease, then further testing for *SMN *mutations should be pursued. Genetic tests now offer quick and reliable *SMN1 *gene copy number testing by using Multiplex ligation-dependent probe amplification (MLPA) or real time PCR. Semiquantitative assays improve diagnostic sensitivity up to 98% [24,44]. If the patient has a single *SMN1 *copy, it is mandatory to sequence the coding region of the undeleted allele to identify the second causative mutation, generally subtle sequence variations, including point mutations, insertions, and deletions. However, in about one third of patients with a typical clinical picture and a single SMN1 copy, the second mutation is not found in *SMN1/SMN2 *coding region. This finding is more common in type III SMA and might be due to the presence of deep intronic mutations, unidentified so far (personal unpublished observation). Finally, sequence analysis of *SMN1 *gene is suggested also in those patients who have a typical clinical picture, 2 SMN1 copies, and are born to consanguineous parents or originate from genetic isolates. Indeed, rare patients homozygous for *SMN1 *subtle mutations have been occasionally reported [45].

Conversely, in a patient with 2 *SMN1*copies, SMA diagnosis, related to *SMN1 *mutations is virtually excluded and other motor neuron disorders such as spinal muscular atrophy with respiratory distress (SMARD1), X-linked spinal muscular atrophy, distal SMA, and juvenile amyotrophic lateral sclerosis should be considered.

If the electrophysiological examination excludes a motor neuron disease the child should be reexamined and must receive additional diagnostic testing considering other disorders.

## Differential diagnosis

In general, the most important differential diagnostic conditions for an infant presenting with hypotonia and/or weakness are congenital myopathies, i.e. myopathies with typical structural or ultrastructural features (rods, cores, central nuclei) on the muscle biopsy, congenital myotonic dystrophy, congenital myasthenic syndromes, metabolic myopathies, congenital disorders of the motor neuron and the peripheral nerve (congenital hypomyelinating neuropathy), as well as non-neuromuscular conditions including genetic syndromes such as, Prader-Willi syndrome, acute hypoxic ischemic encephalopathy, neonatal sepsis and dyskinetic or metabolic conditions.

The most important tools to address these differential diagnostic possibilities beyond the clinical examination and a careful family history are CK determination (note however that the CK can be only moderately elevated in chronic forms of SMA), EMG/nerve conduction studies to discriminate neurogenic conditions and abnormalities of neuromuscular transmission, MRI of the brain, muscle biopsy, and specific genetic or metabolic testing,.

Other inherited motor neuron disorders, not caused by mutation of the *SMN *gene, that present with early weakness should be considered and are listed in table 2. Some clinical symptoms may suggest the diagnosis including joint contractures, distal rather than proximal weakness, diaphragmatic paralysis with early respiratory failure, and pontocerebellar degeneration.

**Table 2:** other forms of SMA not linked to SMN gene

SMA variant	Inheritance/gene	Clinical features
**Scapuloperoneal SMA**	AD 12q24.1-q24.31	Progressive weakness of scapuloperoneal and laryngeal muscles
**SMA with pontocerebellar hypoplasia**	AR *VRK1*	Brainstem and cerebellar hypoplasia, early onset (0-6 mo)
**X-linked infantile SMA with arthogryposis**	X-linked Xp11.3-q11.2 *UBA1*	Contractures, onset at birth or infancy, early death
**SMA with respiratory distress type I**	AR-*IGHMBP2*	Early onset (< 3 mo), eventration of diaphragms, distal weakness, pes equines.
**Congenital distal SMA**	AD12q23-q24	Early onset with contractures, nonprogressive
**Distal SMA-V/CMT2d**	AD 7p15 *GARS*	Distal SMA with upper limb predominance

## Genetic counseling and prenatal diagnosis

Spinal muscular atrophy is one of the most common genetic disorders, with a carrier frequency of about 1/50, and direct carrier testing could be beneficial to community as screening test. Since the most common mutation found in patients is the homozygous absence of *SMN1 *gene, the majority of carriers bear the heterozygous deletion of one *SMN1 *allele. As in the case of second level diagnostic test, carrier testing is based on semiquantitative real time PCR or MLPA. Since the sensitivity of the molecular test is 93-95% [43], it is important to provide all couples performing molecular test with formal genetic counselling for the assessment of residual risk of having a child affected from SMA. The carrier test does not always identify if SMA is present and is not typically performed until the sibling is of childbearing age.

Thus it is imperative that individuals understand the limitations of the molecular testing: subjects who test negative for the search of heterozygous deletion may have two *SMN1 *copies in cis on one chromosome 5, may be carriers of rare subtle mutations, and the occurrence of extremely rare de-novo mutations cannot be ruled out. As (in the case of other) is true for carrier screening programs, SMA testing must be voluntary, performed in adults only, and upon informed consent and assurance of confidentiality [6].

In most cases, carrier testing is requested by siblings of patients or of parents of SMA children and are aimed at gaining information that may help with reproductive planning (prenatal diagnosis or pre-implantation diagnosis). In these cases, we suggest to test at risk individuals first and, in case of testing positive, to analyze also the partner. Thus in case of a request on carrier testing on siblings of an affected SMA infant, a detailed neurological examination should be done and consideration given doing the direct test to exclude SMA.

Prenatal diagnosis should be offered to couples who have previously had a child affected with SMA (recurrence risk 25%); in these cases, antenatal screening by chorionic villi sampling can be carried out between the 11^th ^and 13^th ^week of pregnancy. In all other instances, i.e. relatives of patients, carrier testing is sufficient to reduce markedly the risk of SMA for the offspring. In our opinion, prenatal diagnosis should be offered only when both partners test positive to the carrier screening; however, the clinical severity of a potentially affected foetus cannot be established a priori and the predictive power of *SMN2 *copy number assessment is not sufficient to establish an accurate prognosis. Indeed testing for SMN2 copy number in an affected fetus is problematic when there are 3 copies, as types I, II and III can all result in this setting and thus no prognosis can be established. Having a single copy is rare but is highly predictive of a severe type I baby with a very poor prognosis. A copy number of 2 is typical for a type I phenotype but could be a type II, again making any prognosis difficult. These considerations and facts have impact on neonatal screening, an argument that is growing with the progress in treatment prospective in SMA [6]. The purpose of newborn screening is to identify affected infants prior to the presentation of clinical symptoms. Newborn screening has been an extremely successful program and has improved the quality of life of many children with a variety of disorders. There has now been an expansion of the number of conditions included in many newborn screening panels. The benefits achieved through newborn screening have traditionally referred to the direct benefits to the affected child. However, there are currently a number of disorders screened which do not have a well-defined medical treatment, and this is the case for SMA. While a number of potential therapies are currently in clinical trials for SMA [46-50], their success may depend on identifying individuals as early as possible in order to begin treatment before potentially irreversible neuronal loss. In infants with type I SMA, rapid loss of motor units occurs in the first 3 months and severe denervation with loss of more than 95% of units within 6 months of age [51]. Therefore a very small window for beneficial therapeutic intervention exists in infants with type I SMA. Therapies would need to be administered within the newborn period for maximum benefit which could potentially be accomplished through a newborn screening program for SMA.

## Management

A first consensus for standards of care of SMA has been achieved in recent years [43]. Because of the complexity of medical problems associated with the diagnosis of SMA the primary care plays a central role in coordinating the follow-up and care. The role of follow-up coordination has to be managed by an expert in neuromuscular disorders and in SMA who is able to plan a multidisciplinary intervention that includes pulmonary, gastroenterology/nutrition, and orthopedic care. Pulmonary disease is the major cause of morbidity and mortality in SMA types I and II and may occur in a small proportion of patients with type III. Respiratory failure is caused by a greater involvement of expiratory and intercostal muscles whereas the diaphragm is relatively spared. Swallowing dysfunction and reflux are important contributors to pulmonary morbidity. Patient initially has recurrent chest infections, followed by nocturnal oxygen desaturation, nocturnal hypoventilation, and then daytime hypercarbia [52-54].

Recommendations for respiratory assessment include evaluation of cough effectiveness, observation of breathing, and monitoring gas exchange. Respiratory muscle function tests are indirect measures of cough effectiveness and include peak cough flow, maximal inspiratory pressure, and maximal expiratory pressure. In case of a diagnosis of weak cough effectiveness cough-assist device and oral suction pump is advised. Overnight pulse oximetry with chart recording can be used to screen for nocturnal hypoxemia. Polysomnography with transcutaneous CO2 measurement are useful tools to assess sleep-related hypoventilation.

When nocturnal hypoventilation is detected nocturnal noninvasive ventilation (NIV) must be started with bi-level positive pressure support. NIV can be used as a routine therapy (also in daytime when it is needed) or as a palliative tool. A key goal is to prevent pediatric intensive care unit stays and avoid tracheotomy if possible. NIV should be the first choice to avoid tracheostomy.

Additional therapies are medical or surgical gastroesophageal reflux disease management and nutritional support orally or via a gastrostomy.

In SMA patients muscle weakness resulting in contracture formation, spinal deformity, limited mobility and activities of daily living, and increased risk of pain, osteopenia, and fractures. Infants should have appropriate evaluation for their presenting musculoskeletal and functional deficits. Goals of therapy and surgery depend on functional level and the family's wishes. Whenever possible, walking should be encouraged with appropriate assistive devices and orthotics. Hip subluxation is rarely painful, and there is a high risk of recurrence despite surgical correction. Spinal orthoses may provide postural support but do not prevent curve progression and may impair respiratory effort. Scoliosis surgery appears to benefit patients who survive beyond 2 years of age when curves are severe and progressive and should be performed while pulmonary function is adequate. Over the past few years newer surgical treatments have been developed for the management of severe scoliosis in skeletally immature patients prior to definitive spinal fusion. Growing-rods [55] or Vertical Expandable Prosthetic Titanium Rib (VEPTR) [56] may be used to prevent the progression of the curve in very young children when bracing isn't successful.

## Therapeutic strategies

Actually no cure is available for SMA, and the pathogenesis of the disorder is not completely understood. In the last few years however many progresses in understanding the molecular basis of the disease has been made and different therapeutic approaches are developing [57].

## Pharmacological therapies

Several mechanisms have been targeted in SMA drug trials such as neuroprotective drugs to rescue motorneurons (as riluzole), creatine to improve energy metabolism, and albuterol for its anabolic properties and the molecular effect on *SMN2 *gene expression [58]. Preliminary therapeutic efforts have been dominated by drugs targeting to the modulation of *SMN2 *pre-mRNA splicing, aimed at increasing SMN-fl levels, or to the enhancement of *SMN2 *promoter activity. An alternative therapeutic strategy is based on the use of antisense oligonucleotides (ASOs) targeting the 3' splice site (ss) of exon 8 [59] and inhibiting the function of a negative splicing regulator (E1) within intron 6. The antisense strategy has further evolved by the development of alternative chemistries and through the incorporation of an untethered binding platform for positively acting splicing factors to the SMN2 exon 7 region. This has been accomplished by combining the antisense region with either a covalently bound synthetic peptide or with a non-complementary ESE (exon splicing enhancer) sequence acting as a binding platform for SR proteins (bifunctional RNAs) [60]. Similar to the synthetic RNAs, bifunctional RNAs may be expressed from AAV vectors, leading to increased SMN protein levels in cell-based models [61].

Following extensive high throughput screening of SMN promoter-activating compounds, novel quinazoline derivatives were recently developed, which not only increased SMN in vitro, but also improved the SMA phenotype in the SMNΔ7 mouse model [62,63].

An alternative strategy has been proposed by Mattis et al. (2006) [64]: aminoglycosides induce the read-through of the stop codon located in exon 8 of the SMN-del7 protein, thus elongating the C-terminus and stabilizing the protein *in vitro*. Successful read-through has also been achieved using different scaffolds with acceptable safety profiles as shown by PTC Therapeutics in a clinical trial with cystic fibrosis patients [65].

The group of compounds, histone deacetylase (HDAC) inhibitors, has shown promise in several models of neurodegeneration including SMA mouse models and patients [66]. Positive results have been obtained in murine SMA models with trichostatin A, sodium butyrate, and valproic acid [67-69]. Despite these pre-clinical encouraging results, clinical trials have not given efficacy outcomes using valproate and phenylbutyrate besides good safety profiles [70]. New generation of HDAC inhibitor compounds may hold promise since it has been shown that LBH589 increased SMN levels in cells from patients unresponsive to valproic acid [71], and SAHA administration increased lifespan in an SMA mouse model [72].

## Gene therapy

In addition to possible drug therapy, gene therapy approaches have been evaluated for SMA, using viral vectors to replace *SMN1 *[73]. In a series of experiments, self-complementary AAV8-hSMN was injected at birth intrathecally into the CNS of SMA-like mice, increasing the median life span of affected animals up to 50 days, compared with 15 days for untreated controls [74]. In another study, self-complementary adeno-associated virus (scAAV9) vectors were intravenously injected at postnatal day 1. Survival analysis showed that this treatment rescued 100% of treated animals, increasing life expectancy from 27 to over 340 days (median survival of 199 days). The systemic scAAV9 therapy mediated complete correction of motor function, prevented MN death and rescued the weight loss phenotype close to normal [75]. Fourt et al have shown that self-complementary adeno-associated virus 9 (scAAV9) can infect approximately 60% of motor neurons when injected intravenously into neonatal mice. The scAAV9 was delivered at postnatal day 1 in SMA-like pups and rescued motor function, neuromuscular physiology and life span of affected mice. Later treatment (postnatal day 5) resulted in partial correction of the phenotype, whereas postnatal day 10 treatment had little effect, suggesting a developmental window during which scAAV9 therapy has maximal benefit. Notably the Authors reported extensive scAAV9-mediated motor neuron transduction after injection into a newborn *cynomolgus macaque *demonstrating that scAAV9 crosses the blood-brain barrier in a non-human primate and emphasizing the clinical potential of scAAV9 gene therapy for SMA [76].

Adeno-associated virus (AAV) vectors have also been used to deliver ASOs to the central nervous system by intrathecal infusion. Passini et al (2011) have shown a very efficient transfer rate of oligounucleotides, with increased expression SMN-fl in mice models, and provided evidence that this route of administration has a higher efficiency than systemic delivery [77].

## Stem cell therapy

Stem cell approaches offer promise as a cellular replacement strategy in the treatment of SMA and it is currently receiving considerable attention [78,79]. Cell replacement may be achieved by transplantation of stem cell-derived cells which have undergone maturation in vitro, or by activation of endogenous stem cells in the CNS. Bone marrow transplantation and mesenchimal cells are the only stem cell therapy currently in use, but no experience has been reported in SMA research. Significant progress has been obtained using primary neural stem cells derived from spinal cord, demonstrating improvement of the spinal muscular atrophy phenotype in mice, although this primary source has limited translational applications [80]. In another study these Authors used pluripotent stem cells derived from embryonic stem cells showing the same potential therapeutic effects [81] by injecting ES cell-derived neural cell precursors, into the spinal cord of a relatively severe SMA mouse model. More recently the successful generation of induced pluripotent stem (iPS) cells from patient fibroblast is an important step towards the generation of genetically compatible neurons for stem cell therapy [82].

## Competing interests

The authors declare that they have no competing interests.

## Authors' contributions

AD and EB both wrote down the draft of this paper; EM reviewed the part of the Diagnosis and Management while FDT reviewed the part related to genetic counselling. All authors read and approved the final manuscript.
